# Molecular detection of spotted fever group rickettsiae in ticks parasitizing pet dogs in Shihezi City, northwestern China

**DOI:** 10.1007/s10493-018-00337-1

**Published:** 2019-01-16

**Authors:** Wurelihazi Hazihan, Zhihui Dong, Liping Guo, Kadyken Rizabek, Dzhunysov Askar, Kulmanova Gulzhan, Mahanov Kudaibergen, Akishev Nurlan Kenjebaevich, Tolegen Talgat, Kenesbay Kairullayev, Yuanzhi Wang

**Affiliations:** 1 School of Animal Science and Technology, Shihezi University, Shihezi, 832000 Xinjiang China; 2 School of Medicine, Shihezi University, Xinjiang Uygur Autonomous Region, Shihezi, 832002 China; 3 School of Medicine, Sun yat-sen university, Guangzhou, 510080 China; 4 Department of Food Engineering, Kazakh National Agrarian University, Almaty, 050010 Kazakhstan

**Keywords:** *Rhipicephalus sanguineus* sensu lato, Spotted fever group rickettsiae, Pet dogs, Northwestern China

## Abstract

A total of 178 adult ticks were collected from 32 pet dogs from five veterinary clinics in Shihezi City, Xinjiang Uygur Autonomous Region (XUAR), northwestern China. All the ticks were identified by comprehensive morphological and genetic analyses, and rickettsiae were detected by seven *Rickettsia*-specific genetic markers in the ticks. The ticks collected were identified as *Rhipicephalus sanguineus* sensu lato. Twenty-one of the 178 samples (11.8%) were positive for rickettsiae. Among these, in 13 (61.9%) samples *Candidatus* R. barbariae were identified, in five (23.8%) samples *R. massiliae*, and in three (14.3%) samples *R. conorii*. This study indicates that more attention should be paid to rickettsial infection in pet dogs and their ticks, because the latter may pose an epidemiological risk for tick-borne transmission of rickettsiae to human beings.

**Electronic supplementary material:**

The online version of this article (10.1007/s10493-018-00337-1) contains supplementary material, which is available to authorized users.

## Introduction

Ticks are among the most common ectoparasites of dogs, also involved in the transmission of a number of major diseases in both dogs and humans (Chomel 2011; Dantas-Torres and Otranto 2016). Tick-borne rickettsioses are caused by the spotted fever group rickettsiae (SFGR) of the genus *Rickettsia*, which contains approximately 20 species, and many of which are established or emerging human pathogens (Wood et al. 2012). Besides, more and more new SFGR species have been found across the world, as a result of range expansion of tick populations, changes in landscape and climate, and more accurate diagnostic testing (Trotta et al. 2012; Yunik et al. 2015).

Due to the emerging and re-emerging nature of tick-borne diseases in humans, increasing focus has been placed on research of ticks parasitizing domestic animals (Hiraoka et al. 2005). As in many other countries, in China the dog has become a bonded family member. Regardless the benefits of having pet dogs, pathogens carried by ticks are potentially transmissible to humans, which may represent a health risk, especially to children, elderly people and immunocompromised individuals (Dantas-Torres and Otranto 2014). To date, at least three protozoan (*Theileria, Babesia* and *Hepatozoon*) and five bacterial (*Anaplasma, Ehrlichia, Rickettsia, Coxiella* and *Bartonella*) tick-borne genera have been reported in domestic dogs around the globe (Beck et al. 2009; Brown et al. 2006; Buhariwalla et al. 1996; Camacho et al. 2001; Conrad et al. 1991; Kaewkong et al. 2014; Kamani et al. 2013; Levin et al.^2012^; Mokhtar et al. 2013; Yabsley et al. 2008). In Jiangxi Province, mid-eastern China, *Babesia canis vogeli* and *Babesia gibsoni* were molecularly detected in 780 dog ticks (749 *Rhipicephalus sanguineus*, 16 *Haemaphysalis campaulata* and 15 *Haemaphysalis verticalis*), while all sampled dog ticks were negative for rickettsial agents (Zheng et al. 2017). In Xinjiang Uygur Autonomous Region (XUAR), northwestern China, rickettsial agents were prevalent in ticks infesting both domestic animals and wildlife (Guo et al. 2015, 2016). However, there is limited knowledge on the species of ticks infesting dogs. Here a molecular investigation was carried out for rickettsial agents in pet dog ticks.

## Materials and methods

### Collection and identification of ticks

In 2016–2017, ticks were sampled from 32 pet dogs presented at five veterinary clinics with symptoms of depression, weight loss and anorexia in Shihezi City (483 m above sea level, at 44°268129ʹN 86°0627148ʹE), the northwestern China. The ticks were placed in tubes with 75% ethanol and stored at − 80 °C. All of the ticks were identified morphologically according to previous reports (Filippova 1997; Dantas-Torres et al. 2013a, b). Twenty-nine representative ticks, with 4–6 ticks at each veterinary clinic, were used to analyze tick species and genetic diversity based on partial mitochondrial *16S rRNA* (460 bp), *12S rRNA* (400 bp) and *coxI* (889 bp) gene sequences (Szabó et al. 2005; Chen et al. 2014).

### DNA extraction and molecular detection

After detailed morphological analysis, genomic DNA was extracted from each individual tick using the TIANamp Genomic DNA Kit (TianGen, Beijing, China). The ticks were mechanically crushed twice in sterile water for 15 min and then dried on sterile paper, suspended in 200 µl tissue lysis buffer and 40 µl proteinase K (100 µg/ml), and incubated overnight at 56 °C. The final elution volume was 60 µl. Subsequently, the polymerase chain reaction (PCR) technology was used to detect rickettsial agents with seven genetic markers for DNA fragments [434-, 1332-, 1060-, 488-, 920-, 491-, and 812-bp products of the genes encoding the 17 kilodalton antigen (17-*kDa*), 16S rRNA(*rrs*), citrate synthase (*gltA*), surface cell antigen 1 (*sca1*), PS120-protein-encoding gene (*gene D*), and outer membrane proteins A and B (*ompA* and *ompB*)] (Anstead and Chilton 2013; Chilton 2013; Sekeyova et al. 2001; Wei et al. 2015). (Table 1). *Rickettsia aeschlimannii* from *Rh. turanicus* and double-distilled water were used, respectively, as positive and negative controls (Wei et al. 2015). The PCR products were purified using the TIANgel Midi Purification Kit (TIANGEN, Beijing, China), and then subjected to sequencing (BGI, Shenzhen, China). Phylogenetic analyses were conducted used MEGA version 6.0 based on the *17 kDa*-*rr*s-*gltA*-*ompA*-*ompB*-*gene D* concatenated sequence data of the rickettsiae by Maximum Likelihood (ML) and Neighbor-Joining (NJ) methods (Tamura et al. 2013).

**Table 1:** Primers used in this study for amplifying tick mitochondrial genes and *Rickettsia* spp. in ticks from pet dogs, in Shihezi City, northwestern China

Target	Gen	Primer (reference)	Sequences (5′–3′)	Fragment length (bp)
Tick	*16S rRNA*	*T*-*16S*(F) *T*-*16S*(R) (Chen et al. 2014)	CTGCTCAATGATTTTTTAAATTGCTGTGG CCGGTCTGAACTCAGATCAAGT	460
	*12S rRNA*	*12S*(F) *12S*(R) (Szabó et al. 2005)	AAACTAGGATTAGATACCCTATTATTTTAG CTATGTAACGACTTATCTTAATAAAGAGTG	400
	*coxI*	*TY*-*J*-1,449 *C1*-*N*-2,312 (Chen et al. 2014)	AATTTACAGTTTATCGCCT CATACAATAAAGCCTAATA	889
*Rickettsi*a spp.	*rrs*	*R*-*16S*(F) *R*-*16S*(R) (Anstead and Chilton 2013)	ATCAGTACGGAATAACTTTTA TGCCTCTTGCGTTAGCTCAC	1284
	*17-kDa*	*17*-*kDa*(5F) *17*-*kDa*(3R) *17*-*kDa*(1F) *17*-*kDa*(2R) (Anstead and Chilton 2013)	GCTTTACAAAATTCTAAAAACCATATA TGTCTATCAATTCACAACTTGCCGTT GCTCTTGCAACTTCTATGTT CATTGTTCGTCAGGTTGGCG	434
	*glt*A	*gltA*(F) *gltA*(R) (Anstead and Chilton 2013)	ATGACCAATGAAAATAATAAT ATTGCAAAAAGTACAGTGAACA	1078
	*sca*1	*sca1*(F) *sca1*(R) (Anstead and Chilton 2013)	GGTGATGAAGAAGAGTCTC CTCTTTAAAATTATGTTCTAC	657
	*gene* D	*gene D*(F) *gene D*(R) (Sekeyova et al. 2001; Wei et al. 2015)	CGGTAACCTAGATACAAGTGA TATAAGCTATTGCGTCATCTC	920
	*ompA*	*ompA*(F) *ompA*(R) (Anstead and Chilton 2013)	ATGGCGAATATTTCTCCAAAA AGTGCAGCATTCGCTCCCCCT	530
	*ompB*	*ompB*(F) *OmpB*(R) (Anstead and Chilton 2013)	TACTTCCGGTTACAGCAAAGT AAACAATAATCAAGGTACTGT	812

## Results

**Fig. 1:**
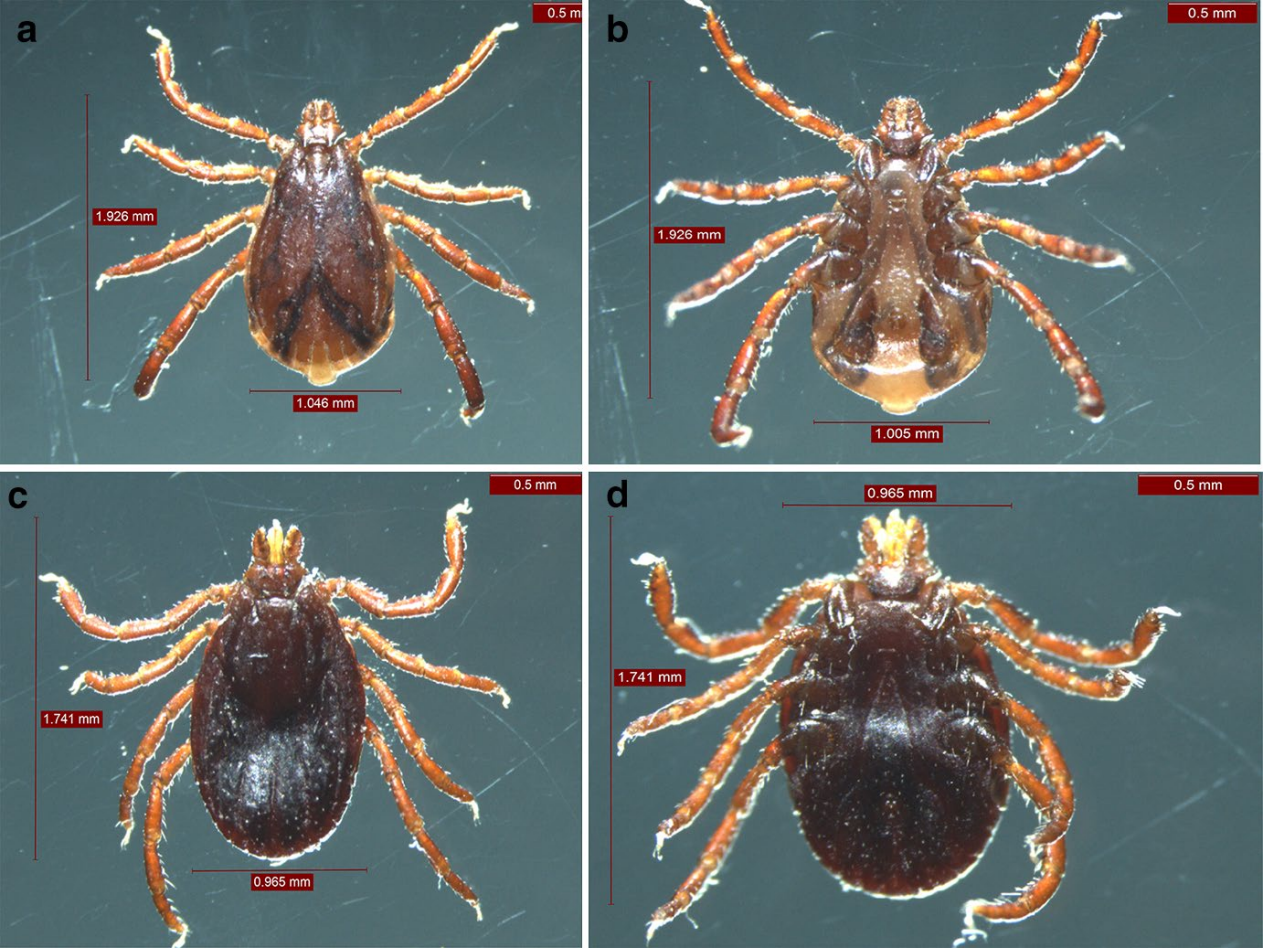
Morphological analysis of *Rhipicephalus sanguineus* sensu lato collected from pet dogs. **a** Male, dorsal; **b** male, ventral; **c** female, dorsal; **d** female, ventral

A total of 178 adult ticks (76 males and 102 females) were collected and morphologically identified as *Rh. sanguineus* sensu lato. (Fig. 1). The sequencing data from the 29 representative ticks confirmed the morphological results based on Basic Local Alignment Search Tool (BLAST) analyses of *16S rRNA, 12S rRNA* and *cox1. Rhipicephalus sanguineus* s.l. in this study had 93.3–93.8% pairwise nucleotide sequence identity to genome sequences of the reference strains *Rh. sanguineus* (GenBank: JX416325) for three genes analyzed. Our data were deposited in the GenBank database (*16SrRNA*: KY069269, *12S rRNA*: KY069270, and *cox1*: KY069271).

**Fig. 2:**
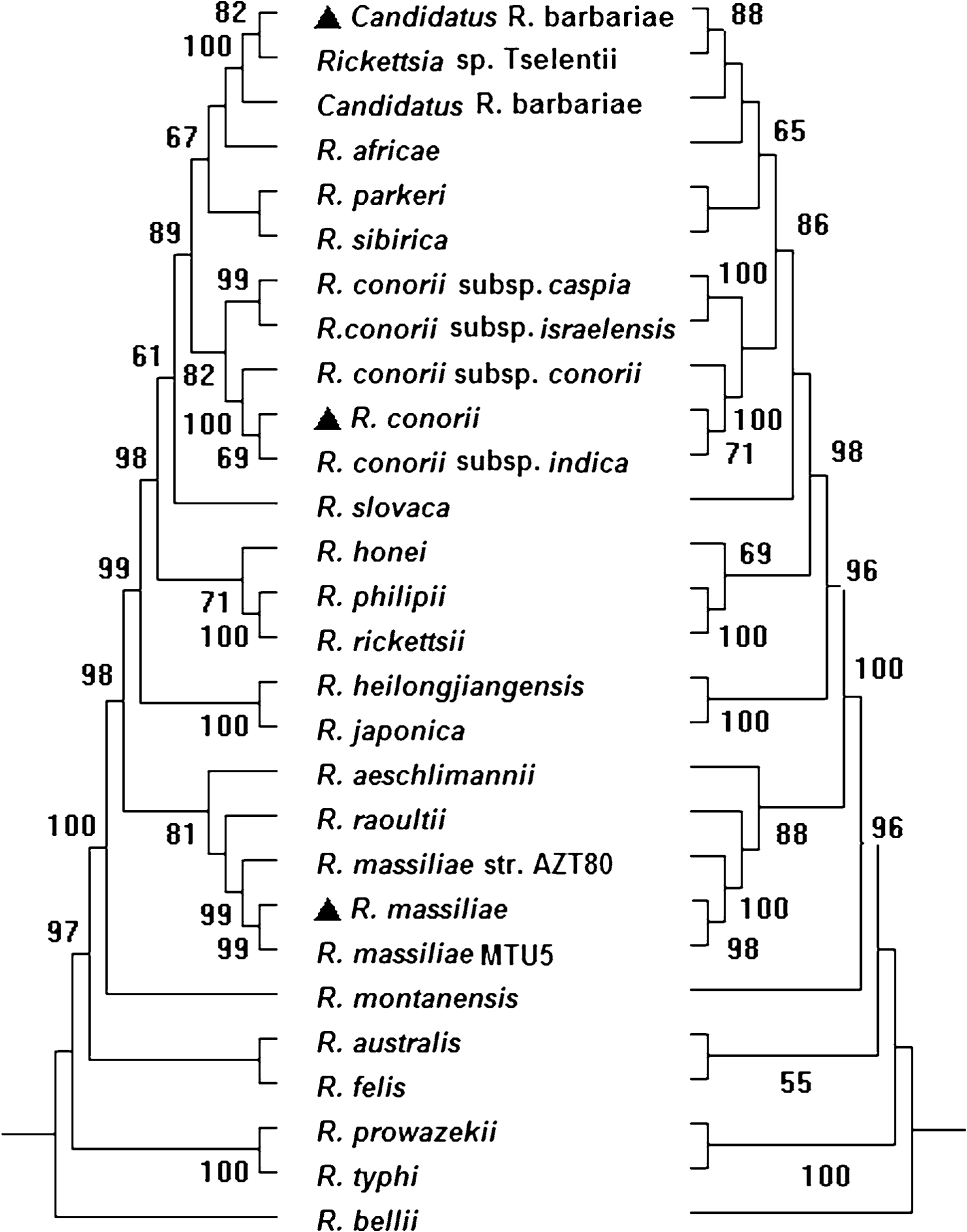
Phylogenetic relationships of *Rickettsia* spp. inferred from *17 kDa*-*rrs*-*gltA*-*ompA*-*ompB*-*gene D* using the Maximum-Likelihood method (left) and Neighbor-Joining method (right). The bootstrap consensus tree inferred from 1000 replicates and bootstrap replicates with value less than 50% were collapsed. Phylogenetic analyses were conducted in MEGA6.0. Rickettsiae obtained in this study were marked as “▲”, and sequences for rickettsia species retrieved from the GenBank database, *Rickettsia bellii* was used as the outgroup (see Additional Table 2).The scale bar represents the inferred substitutions per nucleotide site

Twenty-one of the 178 samples (11.8%) were positive for SFG rickettsiae. Of which, thirteen (61.9%) were identified as *Candidatus* R. barbariae, five (23.8%) as *R. massiliae*, and three (14.3%) as *R. conorii* subsp. *indica*. (Additional Table 2; Fig. 2). *Rickettsia massiliae* and *R. conorii* subsp. *indica* had 99.8–100% and 99.3–100% pairwise nucleotide sequence identities to the corresponding sequences of the reference strains *R. massiliae* MTU5 (GenBank: CP000683) and *R. conorii* str. Malish 7 (GenBank: AE006914) for seven genetic markers, respectively. *Candidatus* R. barbariae in dog ticks showed 100% pairwise nucleotide sequence identity to the corresponding sequences of *Candidatus* R. barbariae in the flea *Vermipsylla alakurt* (according to the seven genetic markers, in GenBank: KT284715, KU645283, KT284716, KU645284, KT284717, KT284718, KU645286, respectively). Detailed similarities of the sequences in this study are shown in Additional Table 1. All the sequences of *Rickettsia* spp. obtained in this study were deposited in GenBank [17 *kDa*: KY069262–KY069264; *rrs*: KY069266–KY069268; *gltA*: KY069259–KY069261; *sca1*: KY069254–KY069255, KY069265; *ompA*: KY069256–KY069258; *ompB*: KY069248–KY069250; *gene D*: KY069251–KY069253].

## Discussion

In the present study, ticks collected from pet dogs were used to identify rickettsial agents in Shihezi City, northwestern China. *Candidatus* R. barbariae, *R. conorii* subsp. *indica* and *R. massiliae* were molecularly detected. Importantly, these rickettsial agents were shown to be present both in pet dog ticks (reported here) and in sheep ticks (Guo et al 2016), which data raise both veterinary and public health concerns in northwestern China.

*Candidatus* R. barbariae was originally reported from *Rhipicephalus bursa* ticks in Portugal (de Sousa et al. 2006), and later confirmed and characterized by five genetic markers (*gltA, ompA, ompB, sca4* and *rrs*) from *Rh. turanicus* in Italy (Mura et al. 2008). Subsequently, *Candidatus* R. barbariae was also detected in *Rh. turanicus* from Cyprus and in *Rh. sanguineus* from Israel (Chochlakis et al. 2012; Waner et al. 2014). In 2016, our investigation revealed that *Candidatus* R. barbariae is present in *Vermipsylla alakurt* fleas and *Rh. turanicus* ticks from grazing sheep (Guo et al. 2016; Zhao et al. 2016). Here, molecular evidence of *Candidatus* R. barbariae is provided in pet dog ticks (*Rh. sanguineus* s.l.).

The other two *Rickettsia* species, *R. conorii* subsp. *indica* and *R. massiliae*, had lower rates of positivity [1.7% (3/178) and 2.8% (5/178), respectively] compared to the data from grazing sheep (Wei et al. 2015; Guo et al. 2016), which might be explained by differences in tick numbers per host, as well as by varying susceptibility to rickettsiae among host species. To the best of our knowledge, however, the clinical cases were caused by *R. conorii* subsp. *indica* and *R. massiliae* (Cavagnaro et al. 2008; Vitale et al. 2006). Although there is no documented clinical case of rickettsia infection from pet dog ticks in China to date, more measures should be carried out to prevent its risk to dog owners, taking into account the synanthropic nature of *Rh. sanguineus* s.l. A diversity of tick-borne pathogens, including *Anaplasma, Babesia, Borrelia, Ehrlichia* and *Theileriai* spp. has recently been molecularly detected in Russia (Livanova et al. 2018). This, together with the present findings, draw the attention to not-yet known risks associated with tick-borne rickettsiae in several regions of Asia.

## Conclusions

Three SFGR members, the *R. conorii* subsp. *indica, Candidatus* R. barbariae and *R. massiliae*, were molecularly detected in *Rh. sanguineus* s.l. ticks from pet dogs in Shihezi City, northwestern China. The study expands the range of tick-borne pathogens in pet dog ticks in Central Asia. Effective measures should be taken into consideration to prevent tick-borne transmission of rickettsiae to human beings.

## Electronic supplementary material

Below is the link to the electronic supplementary material.

Supplementary material 1 (DOCX 29 KB)

Supplementary material 2 (DOC 72 KB)

## References

[CR1] Anstead CA, Chilton NB (2013). A novel *Rickettsia* species detected in vole ticks (*Ixodes angustus*) from Western Canada. Appl Environ Microbiol.

[CR2] Beck R, Vojta L, Mrljak V, Marinculić A, Beck A, Živičnjak T, Cacciò SM (2009). Diversity of *Babesia* and *Theileria* species in symptomatic and asymptomatic dogs in Croatia. Int J Parasitol.

[CR3] Brown GK, Canfield PJ, Dunstan RH, Roberts TK, Martin AR, Brown CS, Irving R (2006). Detection of *Anaplasma platys* and *Babesia canis vogeli* and their impact on platelet numbers in free-roaming dogs associated with remote Aboriginal communities in Australia. Aust Vet J.

[CR4] Buhariwalla F, Cann B, Marrie TJ (1996). A Dog-Related Outbreak of Q Fever. Clin Infect Dis.

[CR5] Camacho AT, Pallas E, Gestal JJ, Guitián FJ, Olmeda AS, Goethert HK, Telford SR (2001). Infection of dogs in north-west Spain with a *Babesia microti*-like agent. Vet Rec.

[CR6] Cavagnaro CS, Brady KA, Siegel C (2008). Fever After International Travel. Clin Pediatr Emerg Med.

[CR7] Chen Z, Li Y, Ren Q, Luo J, Liu Z, Zhou X, Liu G, Luo J, Luo J, Yin Het (2014). *Dermacentor everestianus* Hirst, 1926 (Acari: Ixodidae): phylogenetic status inferred from molecular characteristics. Parasitol Res.

[CR8] Chilton CAANB (2013). A novel rickettsia species detected in vole ticks (*Ixodes angustus*) from western Canada. Appl Environ Microbiol.

[CR9] Chochlakis D, Ioannou I, Sandalakis V, Dimitriou T, Kassinis N, Papadopoulos B, Tselentis Y, Psaroulaki A (2012). Spotted fever group rickettsiae in ticks in cyprus. Microbial Ecology.

[CR10] Chomel B (2011). Tick-borne infections in dogs—An emerging infectious threat. Vet Parasitol.

[CR11] Conrad P, Thomford J, Yamane I, Whiting J, Bosma L, Uno T, Holshuh HJ, Shelly S (1991). Hemolytic anemia caused by *Babesia gibsoni* infection in dogs. J Am Vet Med Assoc.

[CR13] Dantas-Torres F, Otranto D (2014). Dogs, cats, parasites, and humans in Brazil: opening the black box. Parasit Vectors.

[CR14] Dantas-Torres F, Otranto D (2016). Best practices for preventing vector-borne diseases in dogs and humans. Trends Parasitol.

[CR15] Dantas-Torres F, Latrofa MS, Annoscia G, Giannelli A, Parisi A, Otranto D (2013). Morphological and genetic diversity of *Rhipicephalus sanguineus* sensu lato from the new and old worlds. Parasit Vectors.

[CR12] Dantas-Torres F, Capelli G, Giannelli A, Ramos RAN, Lia RP, Cantacessi C, de Caperariis D, De Tommasi AS, Latrofa MS, Lacasella V (2013). Efficacy of an imidacloprid/flumethrin collar against fleas, ticks and tick-borne pathogens in dogs. Parasite Vectors.

[CR16] de Sousa R, Barata C, Vitorino L, Santos-Silva M, Carrapato C, Torgal J, Walker D, Bacellar F (2006). *Rickettsia* sibirica isolation from a patient and detection in ticks, Portugal. Emerg Infect Dis.

[CR17] Filippova NA (1997). Fauna of russia and neighbouring countries. Ixodid ticks of subfamily amblyomminae.

[CR18] Guo LP, Mu LM, Xu J, Jiang SH, Wang AD, Chen CF, Guo G, Zhang WJ, Wang YZ (2015). from marbled polecats, China-Kazakhstan border. Parasit Vectors.

[CR19] Guo LP, Jiang SH, Liu D, Wang sw, Chen CF, Wang YZ (2016). Emerging spotted fever group rickettsiae in Ticks, northwestern China. Ticks Tick Borne Dis.

[CR20] Hiraoka H, Shimada Y, Sakata Y, Watanabe M, Itamoto K, Okuda M, Inokuma H (2005). Detection of rickettsial DNA in ixodid ticks recovered from dogs and cats in Japan. J Vet Med Sci.

[CR21] Kaewkong W, Intapan PM, Sanpool O, Janwan P, Thanchomnang T, Kongklieng A, Tantrawatpan C, Boonmars T, Lulitanond V, Taweethavonsawat P, Chungpivat S, Maleewong W (2014). High throughput pyrosequencing technology for molecular differential detection of *Babesia vogeli. Hepatozoon canis, Ehrlichia canis* and *Anaplasma platys* in canine blood samples. Ticks Tick Borne Dis.

[CR22] Kamani J, Baneth G, Mumcuoglu KY, Waziri NE, Eyal O, Guthmann Y, Harrus S (2013). Molecular detection and characterization of tick-borne pathogens in dogs and ticks from Nigeria. PLoS Negl Trop Dis.

[CR23] Levin ML, Killmaster LF, Zemtsova GE (2012). Domestic dogs (*Canis familiaris*) as reservoir hosts for *Rickettsia conorii*. Vector Borne Zoonotic Dis.

[CR24] Livanova NN, Fomenko NV, Akimov IA, Ivanov MJ, Tikunova NV, Armstrong R, Konyaev SV (2018). Dog survey in Russian veterinary hospitals: tick identification and molecular detection of tick-borne pathogens. Parasit Vectors Nov.

[CR25] Mokhtar AS, Lim SF, Tay ST (2013). Molecular detection of *Anaplasma platys* and *Babesia gibsoni* in dogs in Malaysia. Trop Biomed.

[CR26] Mura A, Masala G, Tola S, Satta G, Fois F, Pirns P, Rolain J-M, Raoult D, Parola P (2008). First direct detection of rickettsial pathogens and a new rickettsia, ‘*Candidatus* R. barbariae’, in ticks from Sardinia, Italy. Clin Microbiol Infect.

[CR27] Sekeyova Z, Roux V, Raoult D (2001). Phylogeny of *Rickettsia spp*. inferred by comparing sequences of ‘*gene* D’, which encodes an intracytoplasmic protein. Int J Syst Evol Microbiol.

[CR28] Szabó MP, Mangold AJ, João CF, Bechara GH, Guglielmone AA (2005). Biological and DNA evidence of two dissimilar populations of the *Rhipicephalus sanguineus* tick group (Acari: Ixodidae) in South America. Vet Parasitol.

[CR29] Tamura K, Stecher G, Peterson D, Filipski A, Kumar S (2013). MEGA6: Molecular evolutionary genetics analysis version 6.0. Mol Biol Evol.

[CR30] Trotta M, Nicetto M, Fogliazza A, Montarsi F, Caldin M, Furlanello T, Solano-Gallego L (2012). Detection of *Leishmania infantum, Babesia canis*, and rickettsiae in ticks removed from dogs living in Italy. Ticks Tick Borne Dis.

[CR31] Vitale G, Mansuelo S, Rolain JM, Raoult D (2006). *Rickettsia massiliae* human isolation. Emerg Infect Dis.

[CR32] Waner T, Keysary A, Eremeeva ME, Din AB, Mumcuoglu KY, King R, Atiya-Nasaqi Y (2014). *Rickettsia africae* and *Candidatus**Rickettsia barbariae* in ticks in Israel. Am J Trop Med Hyg.

[CR33] Wei QQ, Guo LP, Wang AD, Mu LM, Zhang K, Chen CF, Zhang WJ, Wang YZ (2015). The first detection of *Rickettsia aeschlimannii*. and *Rickettsia massiliae* in *Rhipicephalus turanicus* ticks, in northwest China. Parasit Vectors.

[CR34] Wood H, Artsob H (2012). Spotted fever group rickettsiae: a brief review and a Canadian perspective. Zoonoses Public Health.

[CR35] Yabsley MJ, McKibben J, Macpherson CN, Cattan PF, Cherry NA, Hegarty BC, Breitschwerdt EB, O’Connor T, Chandrashekar R, Paterson T, Perea ML, Ball G, Friesen S, Goedde J, Henderson B, Sylvester W (2008). Prevalence of *Ehrlichia canis, Anaplasma platys. Babesia canis* vogeli, *Hepatozoon canis, Bartonella vinsonii berkhoffii*, and *Rickettsia spp*. in dogs from Grenada. Vet Parasitol.

[CR36] Yunik ME, Galloway TD, Lindsay LR (2015). Assessment of prevalence and distribution of spotted fever group rickettsiae in Manitoba, Canada, in the American Dog Tick. *Dermacentor variabilis* (Acari: Ixodidae). Vector Borne Zoonotic Dis.

[CR37] Zhao SS, Li HY, Yin XP, Liu ZQ, Chen CF, Wang YZ (2016). First detection of ‘*Candidatus* R. barbariae’, in the flea *Vermipsylla alakurt*, from north-western China. Parasit Vectors.

[CR38] Zheng W, Liu M, Moumouni PF, Liu X, Efstratiou A, Liu Z, Efstratiou A, Liu Z, Liu Y, Tao H, Guo H, Wang G, Gao Y, Li Z, Ringo AE, Jirapattharasate C, Chen H, Xuan X (2017). First molecular detection of tick-borne pathogens in dogs from Jiangxi, China. J Vet Med Sci.

