# Supplementary material for: Molecular detection of spotted fever group rickettsiae in ticks parasitizing pet dogs in Shihezi City, northwestern China
Source: Exp Appl Acarol. 2019 Jan 16;77(1):73–81. doi: 10.1007/s10493-018-00337-1 (PMC6341051; doi:10.1007/s10493-018-00337-1)
Supplement: Supplementary file 1 — Supplementary material 1 (DOCX 29 KB) [file 10493_2018_337_MOESM1_ESM.docx]

**Additional file 1**

Closest relative sequences to the partial *17*-*kDa*, *rrs*, *gltA*, *ompA*, *ompB*, *sca1* and *gene D* genes of rickettsiae in *Rhipicephalus sanguineus* sensu lato in Shihezi city, in northwestern China. Part A, B and C represent *Rickettsia conorii*, *Candidatus* Rickettsia barbariae, and *Rickettsia massiliae*, respectively.

| Gene | *Rickettsia* (GenBank accession No.) | % Sequence similarity(bp) |
| --- | --- | --- |
| **A** | | |
| *17-kDa* (KY069263) | *Rickettsia conorii* Xinjiang-PS (MF002513) | 100 (434/434) |
|  | *Rickettsia* sp. xinjiang01 (KU364361) | 100 (404/404) |
|  | *Rickettsia conorii* str. Malish 7 (AE006914) | 100 (434/434) |
|  | *Rickettsia conorii* (M28480) | 100 (434/434) |
| *16S （rrs)*  *(*KY069267) | *Rickettsia* sp. xinjiang01 (KU364355) | 100 (1185/1185) |
|  | *Rickettsia conorii* str. Malish 7 (NR_041934) | 100 (1184/1184) |
|  | *Rickettsia conorii* str. Malish 7 (AE006914) | 100 (1185/1185) |
|  | *Rickettsia conorii* isolate PS (MF002584) | 100 (1184/1184) |
| *gltA*  (KY069261) | *Rickettsia conorii* (MF002509) | 100 (1178/1178) |
|  | *Rickettsia* sp*.*xinjiang01 (KU364366) | 100(1076/1076) |
|  | *Rickettsia conorii* str. Malish 7 (AE006914) | 100 (1178/1178) |
|  | *Rickettsia sibirica* subsp. *sibirica* (MF002541) | 99.7 (1174/1178) |
| *ompA* (KY069258) | *Rickettsia conorii* isolate ROD (JN944636) | 100 (534/534) |
|  | *Rickettsia* sp*.* xinjiang01 (KU364365) | 100 (451/451) |
|  | *Rickettsia conorii* (MF002512) | 100 (534/534) |
|  | *Rickettsia conorii* str. Malish 7 (AE006914) | 99.3 (530/534) |
| *ompB*  (KY069249) | *Rickettsia conorii* strain *indian* (AF123726) | 100 (812/812) |
|  | *Rickettsia* sp. xinjiang01 (KU364371) | 99.6 (763/766) |
|  | *Rickettsia conorii* (MF002514) | 100 (812/812) |
|  | *Rickettsia conorii* str. Malish 7 (AE006914) | 99.6 (809/812) |
| *sca1* (KY069255) | *Rickettsia conorii* str. Malish 7 (AE006914) | 99.8 (656/657) |
|  | *Rickettsia* sp. xinjiang01 (KU364363) | 100 (625/625) |
|  | *Rickettsia conorii* (MF002511) | 99.8 (656/657) |
| *gene D* (KY069253) | *Rickettsia conorii* str. Malish 7 (AE006914) | 100 (920/920) |
|  | *Rickettsia* sp. xinjiang01 (KU757303) | 99.9 (896/897) |
|  | *Rickettsia conorii* strain ATCC (AF163005) | 100 (920/920) |
|  | *Rickettsia conorii* strain ATCC VR-141 (AF163008) | 100 (910/910) |
| **B** | | |
| *17-kDa* (KY069264) | *Candidatus* Rickettsia barbariae isolate Xinjiang-YC (MF002507) | 100 (399/399) |
|  | Candidatus Rickettsia barbariae（KT284715) | 100 (363/363) |
|  | *Rickettsia* sp. Tselentii (GU353184) | 100 (399/399) |
| *16S （rrs)*  (KY069268) | Candidatus Rickettsia barbariae (EU272189) | 100 (1284/1284) |
|  | Candidatus Rickettsia barbariae (KU645283) | 100 (1284/1284) |
| *gltA* (KY069260) | Candidatus Rickettsia barbariae (MF002503) | 99.5 (1096/1101) |
|  | Candidatus Rickettsia barbariae (KT284716) | 100 (1045/1045) |
|  | *Rickettsia sibirica* 246 (U59734) | 99.5 (1096/1101) |
|  | *Rickettsia* sp. BJ-90 citrate synthase (AF178035) | 99.5 (1096/1101) |
|  | *Rickettsia parkeri* str. Portsmouth（CP003341） | 99.5 (1095/1101) |
| *ompA* (KY069256) | *Rickettsia* sp. *Tselentii* (EU194445) | 100 (530/530) |
|  | *Candidatus* Rickettsia barbariae (KU645284) | 100 (497/497) |
|  | *Candidatus* Rickettsia barbariae (EU272186) | 100 (530/530) |
|  | *Rickettsia* sp. PoTiRb169 (DQ423366) | 100 (530/530) |
|  | *Candidatus* Rickettsia barbariae (JF700253) | 100 (530/530) |
| *ompB*  (KY069250) | *Candidatus* Rickettsia barbariae (EU272187) | 100 (827/827) |
|  | *Candidatus* Rickettsia barbariae (KT284717) | 100(702/702) |
|  | *Candidatus* Rickettsia barbariae (MF002508) | 100 (827/827) |
| *sca1* (KY069265) | *Candidatus* Rickettsia barbariae (KT284718) | 100 (646/646) |
|  | *Candidatus* Rickettsia barbariae (MF002505) | 100 (646/646) |
| *gene D* (KY069252) | *Candidatus* Rickettsia barbariae (EU272188) | 100 (920/920) |
|  | *Candidatus* Rickettsia barbariae (KU645286) | 100(920/920) |
|  | *Candidatus* Rickettsia barbariae (MF002504) | 100 (920/920) |
| **C** | | |
| *17-kDa* (KY069262) | *Rickettsia massiliae* MTU5 (CP000683) | 100 (434/434) |
|  | *Rickettsia massiliae* strain CABA﹙KT032120﹚ | 99.3 (430/433) |
|  | *Rickettsia massiliae* str. AZT80 (CP003319) | 99.3 (431/434) |
| *16S （rrs)*  (KY069266) | *Rickettsia massiliae* MTU5 (CP000683) | 100 (1184/1184) |
|  | *Rickettsia massiliae* strain Mtu1 (NR_025919) | 100 (1184/1184) |
|  | *Rickettsia massiliae* str. AZT80﹙CP003319 ﹚ | 99.9 (1183/1184) |
| *gltA* (KY069259) | *Rickettsia massiliae* MTU5 (CP000683) | 100 (1178/1178) |
|  | *Rickettsia massiliae* MTU1 (U59719) | 100 (1178/1178) |
|  | *Rickettsia massiliae* str. AZT80 (CP003319) | 99.8 (1176/1178) |
| *ompA* (KY069257) | *Rickettsia massiliae* MTU5 (CP000683) | 99.8(525/526) |
|  | *Rickettsia massiliae* clone 04 ( KR401146) | 99.8(530/531) |
|  | *Rickettsia massiliae* str. AZT80 (CP003319) | 99.1 (523/528) |
| *ompB* (KY069248) | *Rickettsia massiliae* MTU5 (CP000683) | 100 (812/812) |
|  | *Rickettsia massiliae* (AF123714) | 100 (812/812) |
|  | *Rickettsia rhipicephali* AZT80﹙CP003319﹚ | 99.0(807/815) |
| *sca1* (KY069254) | *Rickettsia massiliae* MTU5 (CP000683) | 100（657/657） |
|  | *Rickettsia massiliae* strain Mtu1（AY355364） | 100（657/657） |
|  | *Rickettsia massiliae* str. AZT80（CP003319） | 99.2(652/657) |
| *gene D* (KY069251) | *Rickettsia massiliae* MTU5 (CP000683) | 100 (914/914) |
|  | *Rickettsia massiliae* (AF163003) | 99.9 (909/910) |
