# Supplementary material for: Molecular detection of spotted fever group rickettsiae in ticks parasitizing pet dogs in Shihezi City, northwestern China
Source: Exp Appl Acarol. 2019 Jan 16;77(1):73–81. doi: 10.1007/s10493-018-00337-1 (PMC6341051; doi:10.1007/s10493-018-00337-1)
Supplement: Supplementary file 2 — Supplementary material 2 (DOC 72 KB) [file 10493_2018_337_MOESM2_ESM.doc]

**Additional Table 2**

GenBank accession numbers of the reference sequences used in the phylogenetic analyses.

| *Rickettsiae* | *17-kDa* | *rrs(16s)* | *gltA* | *ompA* | *ompB* | *geneD* |
| --- | --- | --- | --- | --- | --- | --- |
| *R*. *raoultii* | CP010969 | CP010969 | CP010969 | CP010969 | CP010969 | CP010969 |
| *R*. *rickettsii* | CP006010 | CP006010 | CP006010 | CP006010 | CP006010 | CP006010 |
| *R*. *parkeri* | CP003341 | CP003341 | CP003341 | CP003341 | CP003341 | CP003341 |
| *R*. *heilongjiangensis* | CP002912 | CP002912 | CP002912 | CP002912 | CP002912 | CP002912 |
| *R*. *africae* | CP001612 | CP001612 | CP001612 | CP001612 | CP001612 | CP001612 |
| *R*. *massiliae* MTU5 | CP000683 | CP000683 | CP000683 | CP000683 | CP000683 | CP000683 |
| *R*. *massiliae* str. AZT80 | CP003319 | CP003319 | CP003319 | CP003319 | CP003319 | CP003319 |
| *R*. *slovaca* | CP003375 | CP003375 | CP003375 | CP003375 | CP003375 | CP003375 |
| *Candidatus* R. barbariae | MF002507 | EU272189 | MF002503 | EU194445 | MF002508 | MF002504 |
| *Rickettsia* sp. Tselentii | JF803897 | * | [DQ423369](https://www.ncbi.nlm.nih.gov/nucleotide/90657241?report=genbank&log$=nucltop&blast_rank=11&RID=VFPS5RR8016) | EU194445 | JF803894 | * |
| *R*. *australis* | [CP003338](https://www.ncbi.nlm.nih.gov/nucleotide/378931946?report=genbank&log$=nucltop&blast_rank=68&RID=VFE2G4H7014) | [CP003338](https://www.ncbi.nlm.nih.gov/nucleotide/378931946?report=genbank&log$=nucltop&blast_rank=68&RID=VFE2G4H7014) | [CP003338](https://www.ncbi.nlm.nih.gov/nucleotide/378931946?report=genbank&log$=nucltop&blast_rank=68&RID=VFE2G4H7014) | [CP003338](https://www.ncbi.nlm.nih.gov/nucleotide/378931946?report=genbank&log$=nucltop&blast_rank=68&RID=VFE2G4H7014) | [CP003338](https://www.ncbi.nlm.nih.gov/nucleotide/378931946?report=genbank&log$=nucltop&blast_rank=68&RID=VFE2G4H7014) | [CP003338](https://www.ncbi.nlm.nih.gov/nucleotide/378931946?report=genbank&log$=nucltop&blast_rank=68&RID=VFE2G4H7014) |
| 1. *conorii* subsp.*indica* | * | [MF002584](https://www.ncbi.nlm.nih.gov/nucleotide/1198038886?report=genbank&log$=nucltop&blast_rank=2&RID=VFM2V229014) | [MF002509](https://www.ncbi.nlm.nih.gov/nucleotide/1198038737?report=genbank&log$=nucltop&blast_rank=1&RID=VFNEDWWN016) | [JN182803](https://www.ncbi.nlm.nih.gov/nucleotide/388614719?report=genbank&log$=nucltop&blast_rank=4&RID=VFNS7R72014) | [AF123726](https://www.ncbi.nlm.nih.gov/nucleotide/6969967?report=genbank&log$=nucltop&blast_rank=2&RID=VFNBYG5Z016) | AF163005 |
| *R*. *conorii* subsp. israelensis | * | * | [U59727](https://www.ncbi.nlm.nih.gov/nucleotide/1389974?report=genbank&log$=nucltop&blast_rank=1&RID=VFNP6RC8014) | [DQ649059](https://www.ncbi.nlm.nih.gov/nucleotide/109675293?report=genbank&log$=nucltop&blast_rank=29&RID=VFNS7R72014) | [AF123712](https://www.ncbi.nlm.nih.gov/nucleotide/6969939?report=genbank&log$=nucltop&blast_rank=1&RID=VFN6MD64016) | [AF155058](https://www.ncbi.nlm.nih.gov/nucleotide/13568654?report=genbank&log$=nucltop&blast_rank=20&RID=VFKHE23A01R) |
| 1. *conorii* subsp. *conorii* | AY502121 | * | AY502121 | AY502121 | AY502121 | AY502121 |
| 1. *conorii* subsp. *caspia* | * | [L36100](https://www.ncbi.nlm.nih.gov/nucleotide/535747?report=genbank&log$=nucltop&blast_rank=1&RID=VFM2V229014) | [U59728](https://www.ncbi.nlm.nih.gov/nucleotide/1389972?report=genbank&log$=nucltop&blast_rank=25&RID=VFNEDWWN016) | [KY440249](https://www.ncbi.nlm.nih.gov/nucleotide/1170979899?report=genbank&log$=nucltop&blast_rank=20&RID=VFNS7R72014) | [AF123708](https://www.ncbi.nlm.nih.gov/nucleotide/6969931?report=genbank&log$=nucltop&blast_rank=2&RID=VFN6MD64016) | [AF163007](https://www.ncbi.nlm.nih.gov/nucleotide/16506169?report=genbank&log$=nucltop&blast_rank=15&RID=VFKHE23A01R) |
| *R*. *sibirica* | [MF002549](https://www.ncbi.nlm.nih.gov/nucleotide/1198038831?report=genbank&log$=nucltop&blast_rank=1&RID=VFGVZWW4014) | [HM050271](https://www.ncbi.nlm.nih.gov/nucleotide/296409972?report=genbank&log$=nucltop&blast_rank=3&RID=VFJM8NXM014) | [U59734](https://www.ncbi.nlm.nih.gov/nucleotide/1390012?report=genbank&log$=nucltop&blast_rank=5&RID=VFH8XWHY01R) | [AF179365](https://www.ncbi.nlm.nih.gov/nucleotide/9789175?report=genbank&log$=nucltop&blast_rank=6&RID=VFGZKT3D01R) | * | [AF155057](https://www.ncbi.nlm.nih.gov/nucleotide/13568652?report=genbank&log$=nucltop&blast_rank=3&RID=VFKHE23A01R) |
| *R*. *montanensis* | AY355358 | AY355358 | AY355358 | AY355358 | AY355358 | AY355358 |
| *R*. *aeschlimannii* | * | [NR_026042](https://www.ncbi.nlm.nih.gov/nucleotide/219846451?report=genbank&log$=nucltop&blast_rank=1&RID=VFJHHUHU016) | [HM050285](https://www.ncbi.nlm.nih.gov/nucleotide/296409932?report=genbank&log$=nucltop&blast_rank=84&RID=VFH8XWHY01R) | HM050286 | HM050278 | [AF163006](https://www.ncbi.nlm.nih.gov/nucleotide/16506167?report=genbank&log$=nucltop&blast_rank=2&RID=VFKYM8GC016) |
| *R*. *honei* | AF060706 | [NR_025967](https://www.ncbi.nlm.nih.gov/nucleotide/219846376?report=genbank&log$=nucltop&blast_rank=48&RID=VFJM8NXM014) | [U59726](https://www.ncbi.nlm.nih.gov/nucleotide/1390018?report=genbank&log$=nucltop&blast_rank=2&RID=VFHKXYNF01R) | AF018076 | AF123711 | [AF163004](https://www.ncbi.nlm.nih.gov/nucleotide/16506163?report=genbank&log$=nucltop&blast_rank=19&RID=VFKHE23A01R) |
| *R*. *felis* | [CP000053](https://www.ncbi.nlm.nih.gov/nucleotide/67003925?report=genbank&log$=nuclalign&blast_rank=65&RID=VFE2G4H7014) | [CP000053](https://www.ncbi.nlm.nih.gov/nucleotide/67003925?report=genbank&log$=nuclalign&blast_rank=65&RID=VFE2G4H7014) | [CP000053](https://www.ncbi.nlm.nih.gov/nucleotide/67003925?report=genbank&log$=nuclalign&blast_rank=65&RID=VFE2G4H7014) | [CP000053](https://www.ncbi.nlm.nih.gov/nucleotide/67003925?report=genbank&log$=nuclalign&blast_rank=65&RID=VFE2G4H7014) | [CP000053](https://www.ncbi.nlm.nih.gov/nucleotide/67003925?report=genbank&log$=nuclalign&blast_rank=65&RID=VFE2G4H7014) | [CP000053](https://www.ncbi.nlm.nih.gov/nucleotide/67003925?report=genbank&log$=nuclalign&blast_rank=65&RID=VFE2G4H7014) |
| *R*. *typhi* | [CP003398](https://www.ncbi.nlm.nih.gov/nucleotide/380759471?report=genbank&log$=nucltop&blast_rank=82&RID=VFE2G4H7014) | [CP003398](https://www.ncbi.nlm.nih.gov/nucleotide/380759471?report=genbank&log$=nucltop&blast_rank=82&RID=VFE2G4H7014) | [CP003398](https://www.ncbi.nlm.nih.gov/nucleotide/380759471?report=genbank&log$=nucltop&blast_rank=82&RID=VFE2G4H7014) | * | [CP003398](https://www.ncbi.nlm.nih.gov/nucleotide/380759471?report=genbank&log$=nucltop&blast_rank=82&RID=VFE2G4H7014) | [CP003398](https://www.ncbi.nlm.nih.gov/nucleotide/380759471?report=genbank&log$=nucltop&blast_rank=82&RID=VFE2G4H7014) |
| *R*. *prowazekii* | [CP014865](https://www.ncbi.nlm.nih.gov/nucleotide/1012290745?report=genbank&log$=nuclalign&blast_rank=1&RID=VFHG9NKZ01R) | [CP014865](https://www.ncbi.nlm.nih.gov/nucleotide/1012290745?report=genbank&log$=nuclalign&blast_rank=1&RID=VFHG9NKZ01R) | [CP014865](https://www.ncbi.nlm.nih.gov/nucleotide/1012290745?report=genbank&log$=nuclalign&blast_rank=1&RID=VFHG9NKZ01R) | * | * | * |
| *R*. *philipii* | CP003308 | CP003308 | CP003308 | CP003308 | CP003308 | CP003308 |
| *R*. *bellii* | CP000087 | CP000087 | CP000087 | * | CP000087 | CP000087 |

**Note:**

Partial genes were not included in this research because their missing or not appropriate (*).
